# Supratentorial extra-axial *RELA* fusion-positive ependymoma misdiagnosed as meningioma by intraoperative histological and cytological examinations: a case report

**DOI:** 10.1186/s13256-022-03555-9

**Published:** 2022-08-20

**Authors:** Mayumi Akaki Nagayasu, Tsuyoshi Fukushima, Fumitaka Matsumoto, Hideo Takeshima, Yuichiro Sato, Hiroaki Kataoka

**Affiliations:** 1 Clinical Laboratory, University of Miyazaki Hospital, 5200 Kihara, Kiyotake, Miyazaki, Miyazaki 889-1692 Japan; 2 Section of Oncopathology and Regenerative Biology, Department of Pathology, Faculty of Medicine, University of Miyazaki, Miyazaki, Japan; 3 Department of Neurosurgery, Faculty of Medicine, University of Miyazaki, Miyazaki, Japan; 4 Department of Diagnostic Pathology, University of Miyazaki Hospital, Miyazaki, Japan

**Keywords:** Dura-attached supratentorial extra-axial ependymoma, Intraoperative diagnosis, Meningioma, Anaplastic ependymoma, *RELA* fusion-positive ependymoma

## Abstract

**Background:**

Dura-attached supratentorial extra-axial ependymoma is a very rare type of tumor, with only nine reported cases. Preoperative diagnosis of dura-attached supratentorial extra-axial ependymoma is difficult and often radiologically misdiagnosed as a meningioma. We report a case of dura-attached supratentorial extra-axial ependymoma that was misdiagnosed using intraoperative histological and cytological examinations.

**Case presentation:**

A 26-year-old Japanese man with headache and nausea was referred to our medical facility. Magnetic resonance imaging revealed a cystic mass of 70 × 53 × 57 mm in the left temporoparietal lobe. A peritumoral band with hyperintensity on T2-weighted imaging was observed at the periphery of the lesion, suggesting an extra-axial lesion with no apparent connection to the ventricle. A dural tail sign was also noted on the gadolinium-enhanced T1-weighted image. Preoperative clinical diagnosis was meningioma. Proliferated tumor cells in sheets with intermingled branching vessels were observed in the frozen tissue. Perivascular rosettes were inconspicuous, and the tumor cells had rhabdoid cytoplasm. The tumor was intraoperatively diagnosed as a meningioma, suspected to be a rhabdoid meningioma. Perivascular rosettes were evident in the formalin-fixed paraffin-embedded tissues, suggesting ependymoma. The tumor cells had eosinophilic cytoplasm without a rhabdoid appearance. Anaplastic features, such as high tumor cellularity, increased mitotic activity, microvascular proliferation, and necrosis, were observed. Ependymal differentiation was confirmed on the basis of ultrastructural analysis. Molecular analysis detected *C11orf95-RELA* fusion gene. The final diagnosis was *RELA* fusion-positive ependymoma, World Health Organization grade III.

**Conclusion:**

Owing to its unusual location, dura-attached supratentorial extra-axial ependymomas are frequently misdiagnosed as meningiomas. Neuropathologists should take great precaution in intraoperatively diagnosing this rare subtype of ependymoma to avoid misdiagnosis of the lesion as other common dura-attached tumors.

## Background

Ependymoma is a glial tumor in the central nervous system and corresponds to World Health Organization (WHO) grade II category [1]. Anaplastic ependymoma is histologically a higher-grade tumor than classic ependymoma, corresponding to WHO grade III category, characterized by features such as a high nuclear-to-cytoplasmic (N/C) ratio, elevated mitotic counts, high cell density, widespread microvascular proliferation, and necrosis [2]. However, a definite association between histological grade and biological behavior/survival of ependymomas has not been established [1, 2].

Molecular classification of tumor has been proposed as a prognostic tool for ependymal tumors [3]. Poor prognosis is mainly associated with supratentorial classic/anaplastic ependymomas with *RELA* fusion gene [3]. *RELA* fusion-positive ependymomas have become a distinct entity in the classification of ependymal tumors since 2006 [4], and are now classified as grade II or III, depending on histopathological anaplastic features. Recent studies have shown that L1CAM immunohistochemistry can be an alternative for *RELA* fusion gene detection [5].

Ependymomas usually arise in or near the ventricular system [1]. Intracranial extra-axial ependymoma is a rare tumor, and to date, only 27 cases have been reported. Among them, 21 were confirmed as supratentorial extra-axial ependymomas (SEAEs), 9 of which were reported as SEAE attached to the dura mater (Table 1) [6–13]. Dura-attached SEAEs are often preoperatively misdiagnosed as meningiomas on the basis of radiological findings (Table 1).

**Table 1:** Summary of reported cases of supratentorial extra-axial ependymomas (SEAEs) with dural attachment, including the present case

	Authors	Age (years), sex	Location/preoperative tumor size (cm)	Preoperative diagnosis (intraoperative diagnosis)	Surgery	Histology/WHO grade	Adjuvant treatment	Postoperative follow-up months	Comment
1	Hanchery *et al*. [6]	29, M	Interhemisphere/6.5 × 5.5 × 7.5	Meningioma	TR	Classical Ep/II	RT, CT	NA	
2	Hayashi *et al*. [7]	13, M	Rt occipital-parietal/NA	Cystic meningioma Meningeal sarcoma	GTR	Clear-cell Ep/II	None	3 NR	
3	Youkilis *et al*. [8]	20, M	Lt parafalcine/5.0 × 3.0 × 3.2	Meningioma	GTR	Clear-cell Ep/III	None	12 NR	Dural tail sign (+)
4	Salunke *et al*. [9]	43, F	Lt posterior one-third parasagittal/7.2 × 5.9 × 4	Meningioma (meningioma, favor)	GTR	Classical Ep/II	RT	6 NR	
5	Nambirajan *et al*. [10]	9, F	Rt frontal parafalcine/NA	Meningioma Hemangiopericytoma	GTR	Anaplastic, focal clear-cell, Ep/III	RT	6 NR	*C11orf95-RELA* fusion gene (+)
6	Yang *et al*. [11]	47, M	Lt middle-third parafalcine/3.8 × 3.2 × 2.1	Meningioma	GTR	Anaplastic Ep/III	RT	53 NR	Preoperative gamma knife radiosurgery
7	Yang *et al*. [11]	30, M	Rt temporal/3.8 × 3.0 × 3.5	Glioma	NTR GTR	Anaplastic Ep/III	None RT	3-5 R 28 NR	Two lesions
			Rt occipital/6.0 × 5.6 × 6.5	Glioma	GTR	Anaplastic Ep/III	RT	26 NR	
8	Satyarthee *et al*. [12]	9, F	Rt middle-third parafalcine/8.6 × 6.0 × 5.4	Meningioma	GTR	Anaplastic Ep/III	RT	16 NR	
9	Karthigeyan *et al*. [13]	33, F	Rt front-parietal/NA	Meningioma Gliosarcoma Hemangiopericytoma	GTR	Anaplastic Ep/III	RT	12 NR	Dural tail sign (+)
10	Present case	26, M	Lt parietal-temporal/7.0 × 5.3 × 5.7	Meningioma (meningioma)	NTR GTR	Anaplastic Ep/III	RT	12 R 48 R 52 DOD	*C11orf95-RELA* fusion gene (+) Dural tail sign (+)

*M* male, *F* female, *Lt* left, *Rt* right, *NA* not available, *TR* total resection, *GTR* gross total resection, *NTR* near total resection, *Ep* ependymoma, *RT* radiotherapy, *CT* chemotherapy, *NR* no recurrence, *R* recurrence, *DOD* death of disease

Here, we present a case of dura-attached SEAE that was initially misdiagnosed as meningioma on the basis of intraoperative histological and cytological findings. Postoperative evaluation of the paraffin-embedded specimens confirmed this case as an anaplastic ependymoma with dural invasion. Ultrastructural analysis supported the ependymal differentiation of tumor cells, and molecular assays detected *C11orf95-RELA* fusion gene.

## Case presentation

A 26-year-old Japanese male presented with severe headache and nausea for approximately 3 weeks. He had a prior episode of suspected convulsion of which magnetic resonance imaging (MRI) revealed a cystic nodular lesion measuring approximately 60 mm in diameter, located in the left parietal lobe of the cerebrum. The patient was administered medication, and symptoms were subsequently relieved. However, 12 days later, he experienced worsened symptoms and was transferred and admitted to the emergency department of the University of Miyazaki Hospital.

**Fig. 1:**
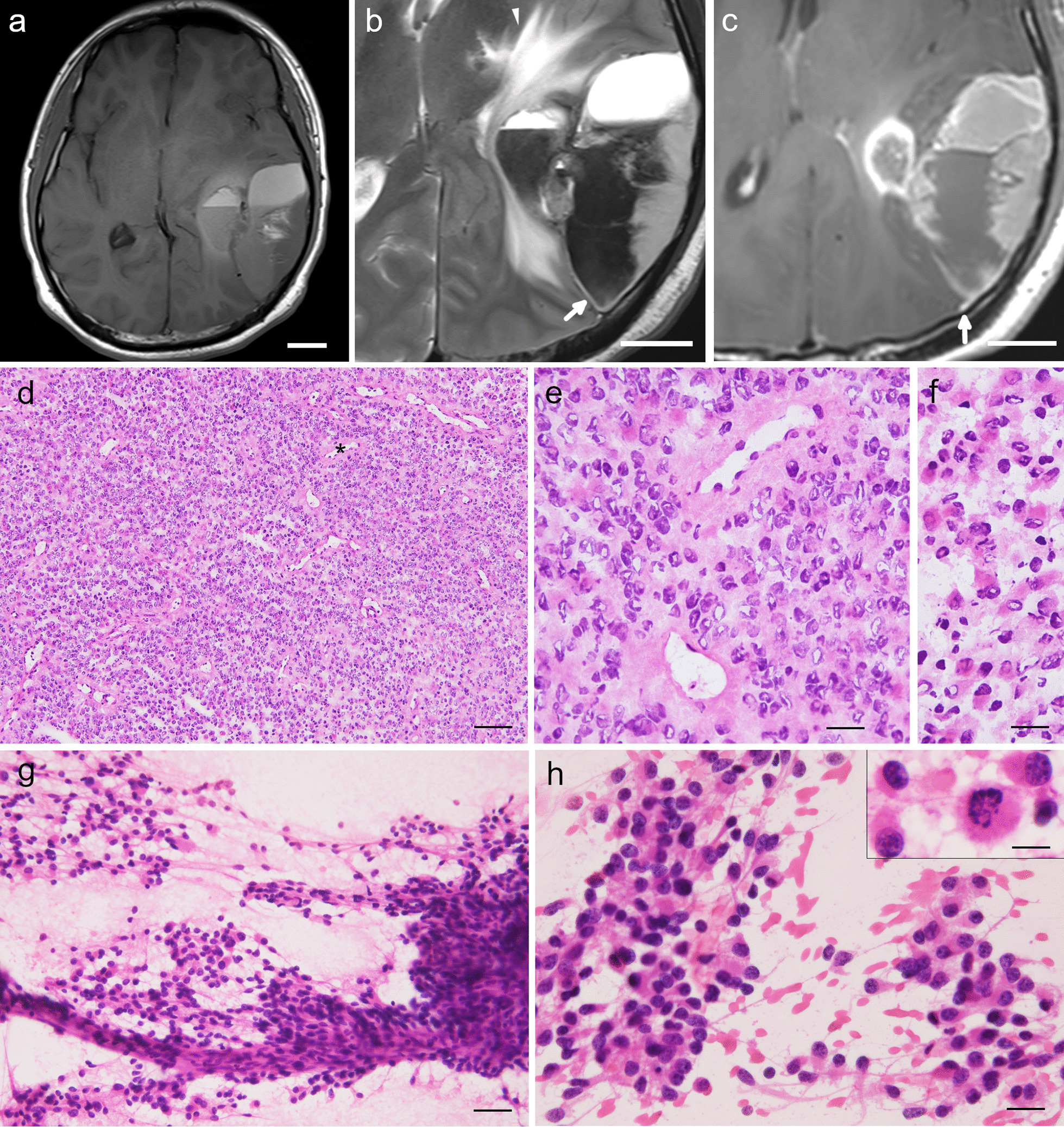
Magnetic resonance imaging, intraoperative histological and cytological findings of the dura-attached supratentorial extra-axial tumor. **a**–**c** Magnetic resonance imaging findings at admission. An axial T1-weighted image showing a cystic mass on left parietal to temporal lobe with hypo- to iso-intensity (**a**). An axial T2-weighted image (**b**) and gadolinium-enhanced T1-weighted image (**c**) showing a heterogeneously hyperintense mass with perifocal edema at the periphery (arrowhead in **b**) and sanguineous material in the cystic spaces. Hyperintense peritumoral band (**b**) and dural tail sign (**c**) are indicated by arrows, suggesting a dura-based extra-axial lesion. **d**–**f**, Intraoperative histological images showing sheet-like proliferation of tumor cells with branching capillary blood vessels (**d**), no apparent fine fibrillary processes in the perivascular anucleate zone (**e** a higher magnification of the blood vessel labeled with an asterisk in **d**), and rhabdoid cytoplasm in tumor cells (**f**). **g**, **h**, Low-magnification (**g**) and high-magnification (**h**) images of an intraoperative cytological specimen. Tumor cells proliferated in irregular clusters close to or distant from blood vessels (**g** squashed specimen). The tumor cells have hyperchromatic nuclei and eosinophilic cytoplasm with fibrillary processes (**h** imprinted specimen). Mitosis is also seen (inset in **h**, squashed specimen). Intraoperative specimens were stained using HE (**d**–**h**). Bars: 2 cm in **a**–**c**, 100 µm in **d**, 25 µm in **e**–**h**, 50 µm in **g**, and 12.5 µm in the inset in **h**

MRI showed a mass of 70 × 53 × 57 mm in the left temporoparietal lobe (Fig. 1a–c). A solid element with slight hypointensity on T1-weighted and hyperintensity on T2-weighted images along the dura mater was noted. Cystic changes were likely associated with the sanguineous fluid. A peritumoral band with hyperintensity on T2-weighted image was observed at the periphery of the lesion, suggesting an extra-axial lesion (Fig. 1b). A dural tail sign was also noted on the gadolinium-enhanced T1-weighted image (Fig. 1c). The lesion appeared to be focally infiltrative to the brain surface, but no apparent connection to the ventricle was observed (Fig. 1a–c). Preoperative clinical diagnosis was meningioma, but hemangiopericytoma and ependymoma were included as differential diagnoses. Left parietotemporal craniotomy was performed. The tumor was an extra-axial mass and had partially infiltrated the dura mater and surface of the brain parenchyma in the left temporal lobe. Small pieces of tumor tissue were removed for intraoperative diagnosis. Under an emergency setting, near-total resection was achieved even if the lesion was in the eloquent area. The patient received postoperative adjuvant radiotherapy (total dose 54 Gy). However, within 1 year postsurgery, the tumor recurred and a second resection surgery was performed. Four years after the first surgery, the tumor metastasized to the spinal cord and the patient eventually died (total postoperative follow-up period was 4 years and 4 months).

## Intraoperative histological/cytological findings

Intraoperative histological specimens were immersed in optimal cutting temperature compound (YUAIKASEI, Japan), fixed in liquid nitrogen, and cryosectioned. Intraoperative cytological specimens were either squashed or imprinted and immediately fixed in 95% ethanol prior to hematoxylin and eosin (HE) staining.

Histological findings of the frozen tumor tissue revealed sheet-like proliferation of tumor cells with branching blood vessels (Fig. 1d). Perivascular rosettes were ambiguous, with no apparent fine fibrillary processes in the perivascular anucleate zones (Fig. 1e). Tumor cells exhibited enlarged hyperchromatic nuclei and condensed eosinophilic cytoplasm, giving a rhabdoid appearance (Fig. 1f). In cytological specimens, irregular clusters of tumor cells were observed in the perivascular and nonperivascular areas (Fig. 1g). The tumor cells had oval hyperchromatic nuclei and uniform eosinophilic cytoplasm with fibrillary processes (Fig. 1h). Mitotic cells were observed (Fig. 1h, inset). Whorl formation or intranuclear cytoplasmic pseudo-inclusions were not evident. Additionally, no apparent microvascular proliferation or necrosis was observed. The lesion was intraoperatively diagnosed as a meningioma, suspected to be a rhabdoid meningioma.

## Histological and immunohistochemical findings for formalin-fixed paraffin-embedded (FFPE) tissues

**Fig. 2:**
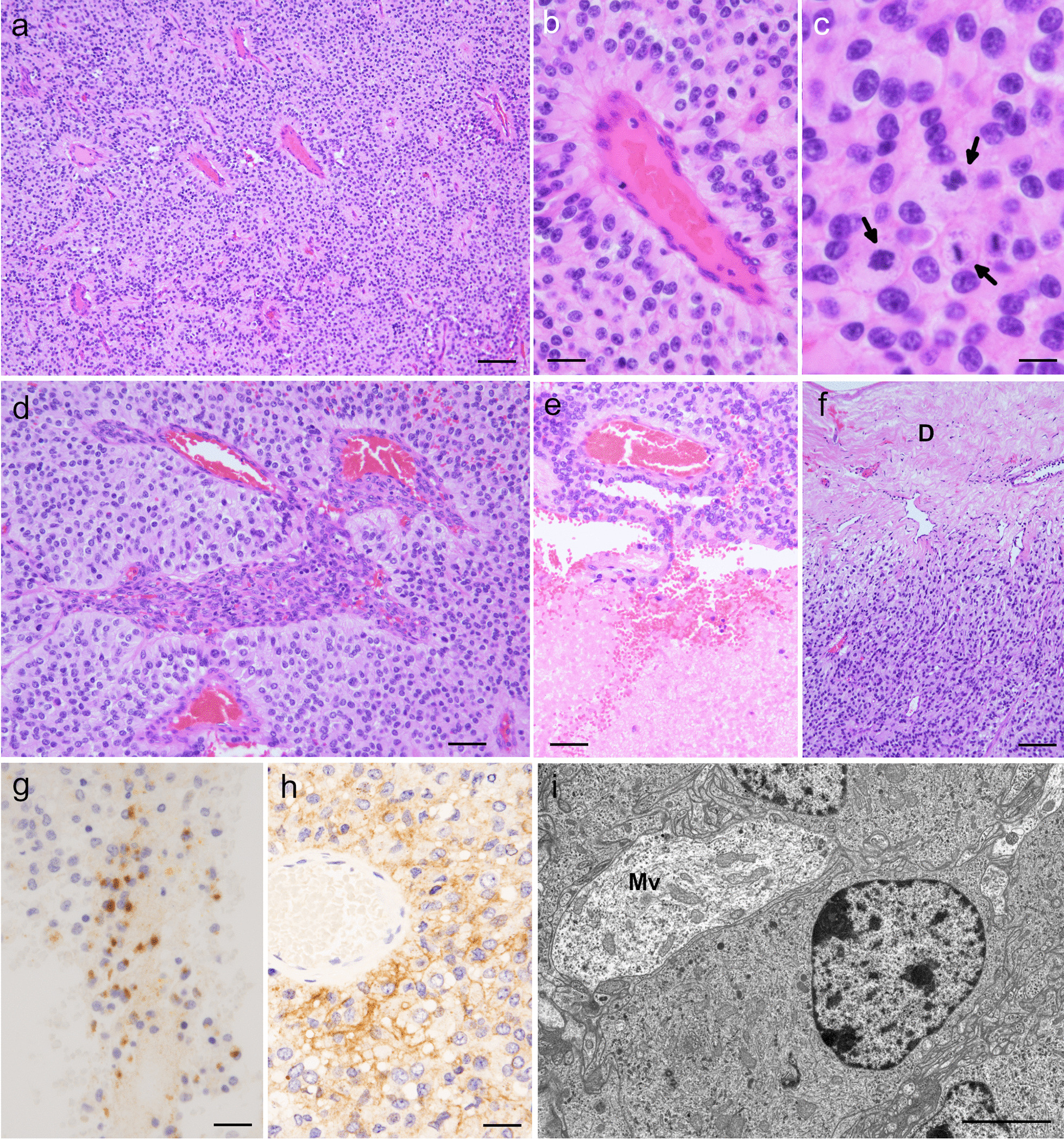
Histological, immunohistochemical, and ultrastructural findings of the dura-attached supratentorial extra-axial tumor. **a**–**f**, Low-magnification (**a** and **d**–**f**) and high-magnification (**b**, **c**) images of the formalin-fixed paraffin-embedded specimen stained using HE showing scattered perivascular rosettes (**a**), prominent fine fibrillary processes in a perivascular anucleate zone (**b**), mitotic cells indicated by arrows (**c**), microvascular proliferation (**d**), necrosis (**e**), and tumor invasion to dura marked by D (**f**). **g**, **h**, Immunohistochemical images with paranuclear dot-like positivity for epithelial membrane antigen (**g**) and membranous positivity for L1 cell adhesion molecule (**h**). **i** Transmission electron micrograph (uranyl acetate–lead citrate staining) exhibiting microvilli marked by Mv in microlumen suggesting ependymal differentiation. Bars: 100 µm in **a** and **f**, 25 µm in **b** and **g**, **h**, 12.5 µm in **c**, 50 µm in **d**, **e**, and 4 µm in **i**

In FFPE tissues, high cellularity was observed, with evidence of tumor cell proliferation and branching vessels (Fig. 2a). Perivascular rosettes were present (Fig. 2a), with distinct fine fibrillary processes in the perivascular anucleate zones (Fig. 2b). The tumor cells exhibited oval hyperchromatic nuclei and eosinophilic cytoplasm with a high N/C ratio (Fig. 2b, c), inconspicuous rhabdoid appearance in the cytoplasm, and high mitotic rates (36 mitotic counts per ten high-power fields) (Fig. 2c). Microvascular proliferation (Fig. 2d), necrosis (Fig. 2e), and dural invasion (Fig. 2f) were observed.

Immunohistochemistry was performed as previously reported [14]. The tumor cells were positive for GFAP, EMA (Fig. 2g), and L1CAM (Fig. 2h). The Ki-67 labeling index was 43% (215/500). Collectively, the lesion was histologically confirmed as an anaplastic ependymoma. Details of the primary antibodies used for the immunohistochemistry are provided in Table 2.

**Table 2:** Details of the primary antibodies used in the study

Antibody	Host	Clone	Dilution	Antigen retrieval	Vendor
GFAP	Mouse	6F2	1:20	ND	Dako (Glostrup, Denmark)
EMA	Mouse	GP1.4	1:100	ND	Dako (Glostrup, Denmark)
Ki-67	Mouse	MIB-1	1:50	100 °C in ERS1, 30 minutes	Dako (Glostrup, Denmark)
L1CAM	Mouse	UJ127	1:1000	100 °C in ERS1, 20 minutes	Sigma-Aldrich (Saint Louis, MO, USA)

The immunohistochemistry was performed by an automated instrument with a Bond Polymer Refine Detection system (Leica Biosystems, New Castle upon Tyne, UK)

*ERS* Bond Epitope Retrieval Solution (Leica Biosystems), *ND* not done

## Ultrastructural findings

A portion of the intraoperative specimen kept at 4 °C was used for ultrastructural analysis [14]. The tumor cells exhibited microlumen formation with microvilli indicating ependymal differentiation, without whorled bundles of intermediate filaments in the cytoplasm (Fig. 2i).

## Molecular findings

**Fig. 3:**
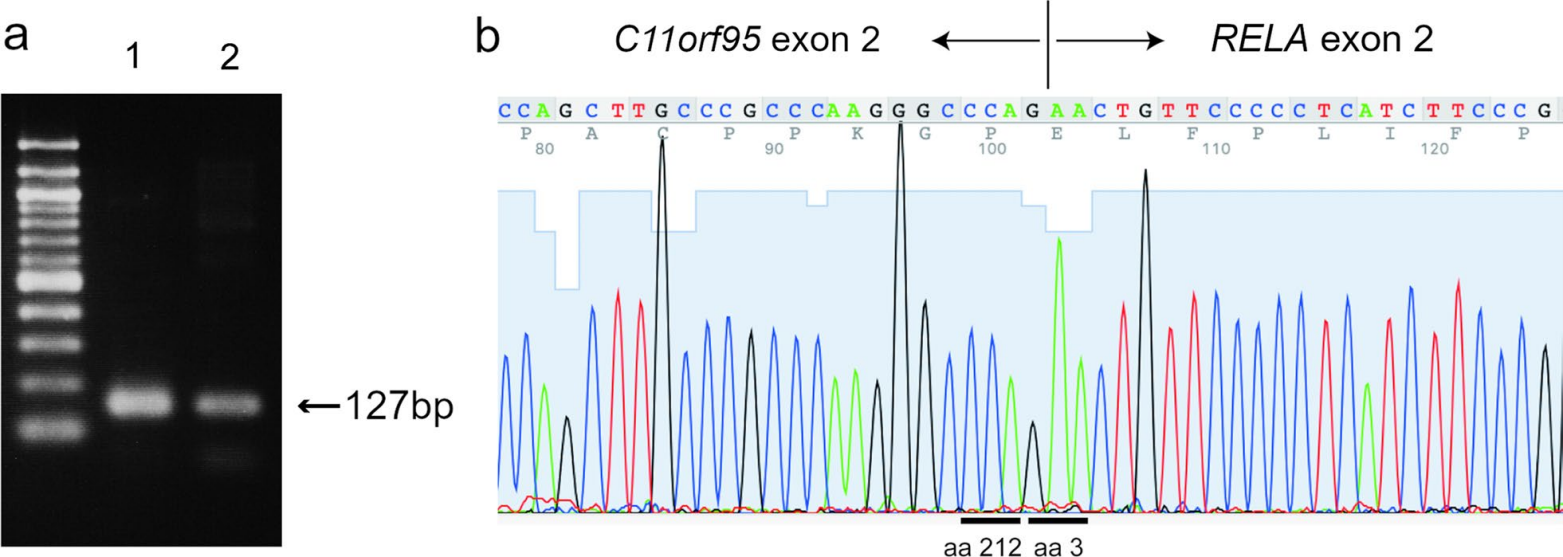
Molecular findings of the dura-attached supratentorial extra-axial tumor. **a**, **b** *C11orf95-RELA* fusion gene detected using polymerase chain reaction assay (**a**; lane 1, the present case; lane 2, positive control) and confirmed using deoxyribonucleic acid sequencing assay (**b**)

To detect *RELA* fusion gene, DNA was extracted from five FFPE sections of 5 μm thickness using a blackPREP FFPE DNA kit (Analitik Jena AG, Jena, Germany) [15]. Polymerase chain reaction (PCR) and DNA sequencing assays were performed, and *C11orf95-RELA* fusion gene was detected (Fig. 3a, b).

The final pathological diagnosis was *RELA* fusion-positive ependymoma, WHO grade III.

## Discussion

We report a case of intraoperatively misdiagnosed rare dura-attached SEAE. The tumor was confirmed as anaplastic ependymoma with dural invasion on the basis of the evaluation of the postoperative histological diagnosis of FFPE specimens. A similar case was reported by Salunke *et al*. [9] as the intraoperative diagnosis “meningioma, favor” (Table 1). However, details about the intraoperative findings were not provided.

An accurate intraoperative diagnosis for dura-attached SEAEs is often challenging, owing to bias by the preoperative information, insufficient time, and lacking ancillary techniques. In our case, to minimize the effect of freezing/drying artifacts during intraoperative diagnosis [16], tissue was snap-fixed in liquid nitrogen. Nevertheless, perivascular rosettes were inconspicuous owing to obscure fine fibrillary processes in the anucleate zones in the intraoperative frozen sections. In the FFPE tissue, perivascular rosettes were evident, and the tumor cells demonstrated eosinophilic cytoplasm without rhabdoid appearance. Electron microscopic examination revealed no apparent whorled bundles of intermediate filaments, usually seen in rhabdoid meningiomas.

*RELA* fusion-positive ependymoma is considered the worst prognosis category of the three molecular subgroups of supratentorial ependymoma [3]. However, a few studies have reported favorable outcomes in patients diagnosed with *RELA* fusion-positive ependymoma and who had undergone with gross total resection [17, 18]. *C11orf95-RELA* fusion gene was previously reported in one SEAE case with a history of 6-month follow-up and no recurrence [10]. The present case with the same fusion gene detected had two recurrences during the postoperative follow-up period of 1 and 4 years, and the patient died 4 months after the second recurrence. Therefore, the biological significance of the *RELA* fusion gene as a prognostic factor remains ambiguous in supratentorial ependymoma.

## Conclusion

We report a case of dura-attached SEAE misdiagnosed as meningioma on the basis of intraoperative diagnosis using frozen tissue sections and cytology. Neuropathologists should take great precaution in intraoperatively diagnosing this rare subtype of ependymoma to avoid misdiagnosis of the lesion as other common dura-attached tumors.

## Data Availability

Not applicable.

## Abbreviations

*RELA*: V-rel avian reticuloendotheliosis viral oncogene homolog A
SEAE: Supratentorial extra-axial ependymoma
*C11orf95*: Chromosome 11 open reading frame 95
WHO: World Health Organization
N/C: Nuclear-to-cytoplasmic
L1CAM: L1 cell adhesion molecule
MRI: Magnetic resonance imaging
Gy: Gray
HE: Hematoxylin and eosin
FFPE: Formalin-fixed paraffin-embedded
GFAP: Glial fibrillary acidic protein
EMA: Epithelial membrane antigen
